# Psychological Impact of the COVID-19 Pandemic on Out-of-Hospital Health Professionals: A Living Systematic Review

**DOI:** 10.3390/jcm10235578

**Published:** 2021-11-27

**Authors:** Raúl Soto-Cámara, Noemí García-Santa-Basilia, Henar Onrubia-Baticón, Rosa M. Cárdaba-García, José Julio Jiménez-Alegre, Ana María Reques-Marugán, María Molina-Oliva, Juan José Fernández-Domínguez, María Paz Matellán-Hernández, Almudena Morales-Sanchez, Susana Navalpotro-Pascual

**Affiliations:** 1 Department of Health Sciences, University of Burgos, 09001 Burgos, Spain; rscamara@ubu.es; 2 Emergency Medical Service of Castilla y León—Sacyl, 47007 Valladolid, Spain; monrubia@saludcastillayleon.es (H.O.-B.); amreques@saludcastillayleon.es (A.M.R.-M.); mmolinao@saludcastillayleon.es (M.M.-O.); mmatellanh@saludcastillayleon.es (M.P.M.-H.); amorales@saludcastillayleon.es (A.M.-S.); 3 Nursing Department, Faculty of Nursing, University of Valladolid, 47005 Valladolid, Spain; 4 Emergency Medical Service of Madrid—SUMMA 112, 28045 Madrid, Spain; juljimal@gmail.com (J.J.J.-A.); jjfernandezd@gmail.com (J.J.F.-D.); snavalpotro@gmail.com (S.N.-P.)

**Keywords:** COVID-19, pandemic, health professionals, out-of-hospital, mental health, anxiety, depression, stress, self-efficacy

## Abstract

Health professionals (HPs), especially those working in the front line, have been one of the groups most affected by the COVID-19 pandemic. The objective of this study is to identify the best available scientific evidence on the impact of the COVID-19 pandemic on the mental health of out-of-hospital HPs in terms of stress, anxiety, depression, and self-efficacy. A living systematic review of the literature was designed, consulting the electronic online versions of the CINHAL, Cochrane Library, Cuiden, IBECS, JBI, LILACS, Medline PyscoDoc, PsycoINFO, Scopus, and Web of Science databases in November 2021. Original research was selected, published in either English, Spanish, French, Italian, or Portuguese. In total, 2082 publications were identified, of which 16 were included in this review. The mental health of out-of-hospital HPs was affected. Being a woman or having direct contact with patients showing suspicious signs of COVID-19 or confirmed cases were the factors related to a greater risk of developing high levels of stress and anxiety; in the case of depressive symptoms, it was having a clinical history of illnesses that could weaken their defenses against infection. Stopping unpleasant emotions and thoughts was the coping strategy most frequently used by these HPs.

## 1. Introduction

In December 2019, the Chinese health authorities reported the presence of new cases of atypical pneumonia of non-identified etiology in Wuhan (Hubei, China) [1]. Subsequently, it was confirmed that the causal pathogenic agent was a new betacoronavirus (RNA), sharing phylogenetic similarities with SARS-CoV-1 [2,3], for which reason it was labeled as SARS-CoV-2. Viral transmission between people principally occurs through the air, although on some occasions, it is through conjunctival, nasal, or buccal mucosa and feces [4].

The rapid propagation of cases between continents, together with community transmission in different countries, led the World Health Organization (WHO) to declare the illness caused by the COVID-19 virus as “The Sixth Public Health Emergency of International Concern”, proclaiming a pandemic situation on 11 March 2020, with over 118,000 cases confirmed in 114 countries and 4291 deaths [5,6].

**Figure 1:**
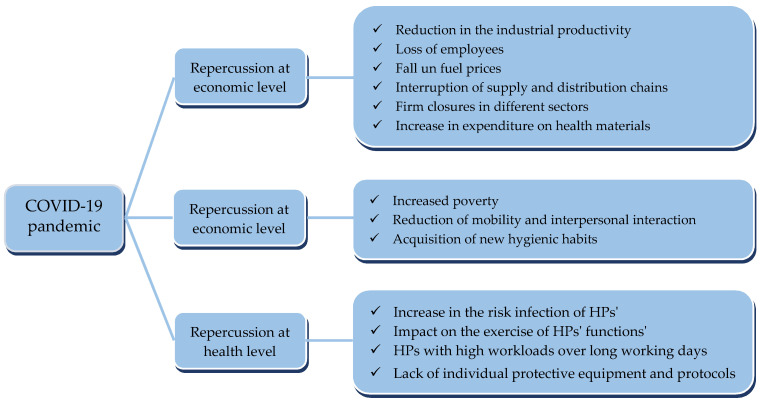
Diagram of the main repercussions of the COVID-19 pandemic.

This situation had important repercussions, to a greater or lesser extent, at economic, social, and health-care levels within all the countries that were affected. At an economic level, there was a significant reduction in industrial productivity, a considerable loss in the number of employees, a fall in fuel prices, the interruption of supply and distribution chains, multiple firm closures in different sectors, and a substantial increase in expenditure on health materials, all of which were framed within volatile and unstable scenarios [7,8,9]. At a social level, as well as the increased poverty arising from the economic changes that were taking place, significant governmental restrictions on the general population were imposed in most countries for the purpose of avoiding or minimizing the spread of the virus. Some of these measures were based on the reduction of mobility and interpersonal interaction, such as strict home confinement and social distancing, whereas others were based on the acquisition of new hygienic habits, such as washing hands or the obligatory use of facemasks [10,11,12]. At the health-care level, the health systems in all countries had to adapt their organization and functioning to the new epidemiological situation in existence. As a consequence, health professionals (HPs) were subjected to high workloads over long working days, considerably reducing their hours of rest, with a lack of approved individual protective equipment and with no clear and defined protocols for action; all of these circumstances increased their risk of infection [13,14,15]. Continuous exposure to this new situation, together with the fear of infecting family members and isolation or social discrimination that they suffered on many occasions, could affect the exercise of their professional functions, considerably reducing their attention span, understanding, and decision-making capabilities [13,16,17]. Despite the above, a study conducted in Singapore showed that the recovery of spontaneous circulation in patients in cardio-respiratory arrest was not lower than before the pandemic [18]. However, some HPs, especially those in the front line, saw that their general well-being had been altered, and they started to show signs of physical and mental exhaustion, high levels of anxiety and depression, other emotional disorders, dysfunctional cognitive reactions, sleep problems, difficulty in interpersonal relations, substances use behaviors, and even post-traumatic stress [19,20,21,22,23]. In this sense, some studies have concluded that nurses entering into direct contact with COVID-19-infected patients have been the HPs at most risk of developing these adverse results during the pandemic [24] (Figure 1).

HPs had to have sufficient levels of self-efficacy and to adopt adequate coping strategies in order to be able to manage this new situation, to avoid the appearance of maladaptive responses, and to reduce the risk of developing post-traumatic stress or other mid-to-long-term associated pathologies [25,26]. According to Bandura, self-efficacy is defined as “the judgments of each individual on his capacity, on the basis of which he will organize and execute his acts in a way that allows them to achieve the desired performance” [27]. On the other hand, the term coping includes the cognitive and behavioral efforts that the person makes to control, reduce, and tolerate the internal and external demands of a certain situation in which their individual resources are exceeded [28]. In a general way, stopping unpleasant emotions and thoughts is the most effective coping strategy for the reduction of stress levels and an increase in positive mental states [26]. However, avoidance behavior is the most widely used strategy, associated with higher levels of stress and the development of post-traumatic symptomology [29,30,31]. Problem-focused strategies are usually correlated with a lower impact on the mental health of HPs [32,33]. During the COVID-19 pandemic, HPs who adopted passive coping strategies presented higher levels of stress, anxiety, and depression. Moreover, perceived social support and active coping strategies were negatively correlated with these variables, which favored compliance with the security protocols against COVID-19 and the adoption of social-distancing measures [34,35,36].

**Figure 2:**
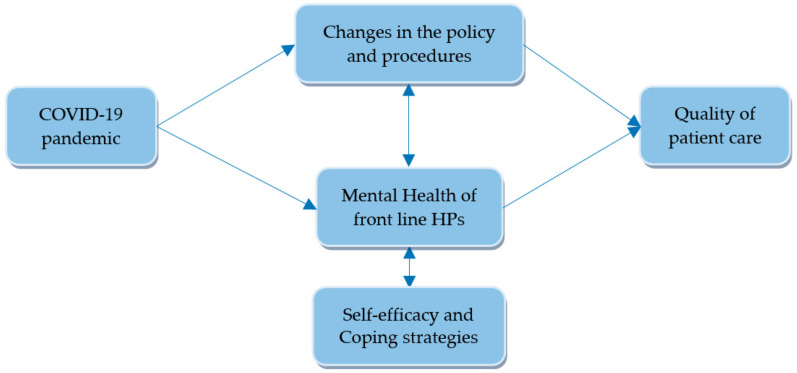
Diagram illustrating the impact of the COVID-19 pandemic on the healthcare system and mental health of front-line HPs.

Emergency medical service (EMS) is in charge of out-of-hospital care for critically ill patients. To face the COVID-19 pandemic, this service had to develop policies and procedures to address the safe caring of patients with suspected or known COVID-19 and a potential increase in the volume of calls. In some countries with low SARS-CoV-2 transmission rates, such as Singapore, EMS use was largely stable during the social distancing and home confinement period compared to previous figures [18]. However, in a study conducted in the United States during the first phase of the pandemic, a general decrease in the number of EMS activations was identified, compared to the prior weeks and the same period in previous years, as well as an increase in the rate of EMS-attended deaths [37]. These results may be due to the fact that patients requiring hospitalization refuse to be transported for fear of being infected by healthcare personnel or other patients. Another study concluded that the decline in EMS use for cardiac arrest, stroke, or time-sensitive illness during the peak of the pandemic was related to patient perception rather than actual case count [38]. In many cases, the HPs from these EMS were first front-line healthcare providers to patients showing suspicious signs of COVID-19 or who were confirmed cases, making them one of the groups of workers most affected by this pandemic [39]. The working conditions of EMS HPs have been particularly vulnerable with respect to the hospital workers. The homogeneous diffusion of the aerosols resulting from a patient´s cough across the entire ambulance, through the ventilation systems, together with the close contact with the patient for longer periods of time, makes these HPs one of the collectives with the highest risk of contracting this infectious disease [39,40]. Indeed, these patients have been transported to hospitals considerably further away from their place of residence due to frequent closures of hospital wards [41]. Based on this, several studies have already assessed how these workers can work in the best possible way when facing a pandemic situation. Like HPs in other areas, the main concerns perceived by out-of-hospital workers have been their moderate degree of training and knowledge about COVID-19, the risk of infecting themselves or their family members, and the lack of personal protective equipment [41,42,43]. These concerns may lead to poorer mental health, resulting in a decreased quality of patient care [44] (Figure 2).

Given the framework of references set out above, the objective of this study is to identify the best available scientific evidence on the impact that the COVID-19 pandemic has had on the mental health of out-of-hospital HPs in terms of stress, anxiety, depression, and self-efficacy.

## 2. Materials and Methods

Following a previously established research protocol, agreed on by the team of researchers, and in accordance with the stipulations presented in the PRISMA declaration (Preferred Reporting Items for Systematic Reviews and Meta-Analyses) [45], a living systematic review of the available scientific literature was designed. To do so, the electronic version of the following databases were consulted in November 2021: Cumulative Index of Nursing and Allied Literature—CINHAL (EBSCOhost, Ipswich, MA, USA), Cochrane Library, Cuiden, Índice Bibliográfico Español en Ciencias de la Salud—IBECS (BVS, Sao Paulo, Brazil), Joanna Briggs Institute—JBI (Ovid, New York, NY, USA), Literatura Latinoamericana y del Caribe en Ciencias de la Salud—LILACS (BVS, Sao Paulo, Brazil), Medline (Pubmed, Bethesda, MD, USA), PyscoDoc (Ovid, New York, NY, USA), PsycINFO (Ovid, New York, NY, USA), Scopus (Elsevier, New York, NY, USA), and Web of Science—WOS (Elsevier, New York, NY, USA). The study protocol has previously been registered at the International Prospective Register of Systematic Reviews PROSPERO, supported by the Centre for Reviews and Dissemination of the University of York, under reference CRD-42021259951.

This living systematic review forms part of a broader project, IMPSYCOVID-19 (Impacto Psicológico de la COVID-19), carried out by the RINVEMER (Red de Investigación en Emergencias prehospitalarias) research group, whose objective is to study the stress, anxiety, depression, and self-efficacy among out-of-hospital HPs in Spain. RINVEMER is a multidisciplinary team composed of 23 members working outside the hospital (physicians, nurses, emergency medical technicians, and psychologists). Among them, 11 members were specifically assigned to the development of this living systematic review.

The search began with the formulation of the following research question by R.S.C., whose clinical response was possible, in PIO (Population-Intervention-Outcome) format [46]: “Has the possible exposure to SARS-CoV-2 during the COVID-19 pandemic (I) affected the mental health (O) of HPs providing health care outside the hospital (P)? From it, the Medical Subject Headings (MeSH), the Descriptors in Health Sciences (DeCS), and synonyms-free text adequate to the objective of the study were identified and combined using the Boolean operators AND and OR. In addition, some of them were truncated in order to include all possible word endings. The initial search strategy was the same for all the databases consulted, adapting it to the particularities of each one of them (Table 1). With the aim of identifying other potentially relevant works that had not previously been recovered, a manual inverse search strategy was proposed, reviewing webpages, sources of grey literature (ProQuest Dissertations and Theses Global, and OpenGrey), as well as bibliographic references cited in the selected studies. All the search strategies in use were developed, reproduced, and checked by three different researchers (R.S.C., R.M.C.G., and S.N.P.) for the purpose of guaranteeing the reliability of the results by comparing those obtained by each of them.

The selected research studies had to meet the following inclusion criteria: (1) being original, (2) based on qualitative and/or quantitative methods, (3) with any methodological design, (4) submitted for peer-review, (5) published in English, Spanish, French, Italian or Portuguese, (6) completed after December 2019, (7) without geographic limitation, (8) with at least the abstract available, and (9) that in their results evaluated the impact of possible exposure to SARS-CoV-2 on levels of stress, anxiety, depression and/or self-efficacy of the HPs who were working in public, private, or voluntary out-of-hospital EMS during the COVID-19 pandemic or identified factors related positively or negatively with these levels or compared them with those obtained in other working environments and/or professional categories. Opinion articles and editorials, studies of low scientific-technical quality, those that did not reply to the posed research question and/or were not in line with the objective of the review, as well as others that, despite including out-of-hospital HPs, contributed no specific data on this subgroup in their results were excluded.

Critical Appraisal Tools from JBI of the University of Adelaide (Australia) [47], considered adequate for the design of the study [48,49], were used to evaluate the scientific–technical quality of the selected articles as well as to determine the extent to which the risk of bias was reduced or eliminated in their design, performance, and/or analysis. These multiple-choice questionnaires have four response options (“yes”, “no”, “unclear”, and “not-applicable”) in a way that a greater number of “yes” responses point to a study of better methodological quality. Each individual study was classified as having a low-, moderate-, or high-risk level of bias based on the number of items answered with “yes”. For qualitative studies (10 items), the methodological quality was considered low, moderate, or high if three or less, four to seven, or more than eight criteria were met, respectively [48]. For cross-sectional studies (8 items), the methodological quality was considered low, moderate, or high if two or less, moderate if three to five, and high if six to eight criteria were met, respectively [49]. High methodological quality was established for the inclusion of studies in the review. Prior to its use, a trial test was carried out in which the reviewers (N.G.S.B., H.O.B., J.J.J.A., A.M.R.M., M.M.O., J.J.F.D., M.P.M.H., and A.M.S.) had to evaluate three articles, subsequently analyzing the degree of concordance between their evaluations.

A standardized data-extraction form was designed in accordance with the JBI [50] recommendations with the purpose of guaranteeing the homogeneity of researchers in the collection of the information as well as facilitating its subsequent analysis and comparison. Based on the research question formulated as well as the inclusion and exclusion criteria considered, the following data were specifically extracted from each selected article: title and principal author, country and year of publication, objective, type of study, place and period undertaken, size and characteristics of the sample, definition of variables under analysis and instruments used, principal results obtained, conclusions of the study and scientific–technical quality. Eight reviewers (N.G.S.B., H.O.B., J.J.J.A., A.M.R.M., M.M.O., J.J.F.D., M.P.M.H., and A.M.S.) previously piloted this data-extraction form on a random sample of three included studies to ensure the agreement among the interpretation of different data items. One group of reviewers (N.G.S.B., H.O.B., J.J.J.A., and J.F.F.D.) extracted data from the included studies using this form, whereas the second one (A.M.R.M., M.M.O, M.P.M.H., and A.M.S.) verified the extracted data.

Three independent reviewers (R.S.C., R.M.C.G., and S.N.P.) screened out possible relevant studies by titles and abstracts, excluding records that did not meet the inclusion criteria. All the works that had initially been identified in each of the databases consulted were included in Mendeley^®^ Reference Manager (Elsevier, New York, US), with the objective of removing duplicates. The final selection of the studies, the evaluation of their methodological quality, and the data-extraction was completed in pairs (N.G.S.B.–H.O.B., J.J.J.A.–J.J.F.D., A.M.R.M.–M.M.O., and M.P.M.H.–A.M.S.), independently with blind reviews, thereby resolving any possible discrepancies through consensus and, in its absence, calling for the participation of a third evaluator (R.M.C.G.). In the face of any doubt or relevant data unavailable in the selected studies, we planned to contact the corresponding author directly, requesting the necessary clarifications.

Because the field of COVID-19 research is moving relatively quickly and new knowledge evidence is continually emerging, an update plan for this systematic review is needed in order to provide convincing evidence for HPs and policymakers. For this, identical search operations will be performed by R.S.C., R.M.C.G., and S.N.P. to identify newly published data. In those databases that have an automatic alert system, these will be configured to provide a feed of new appointments every two weeks. In those others in which automatic alerts are not available, a manual search will be carried out every two weeks. An updated review will be resubmitted when there are relevant changes in the results or when heterogeneity becomes substantial. This systematic review will be maintained in living mode for at least 12 months from publication, although it could be extended at 6-monthly intervals if further evidence is published regularly.

## 3. Results

**Figure 3:**
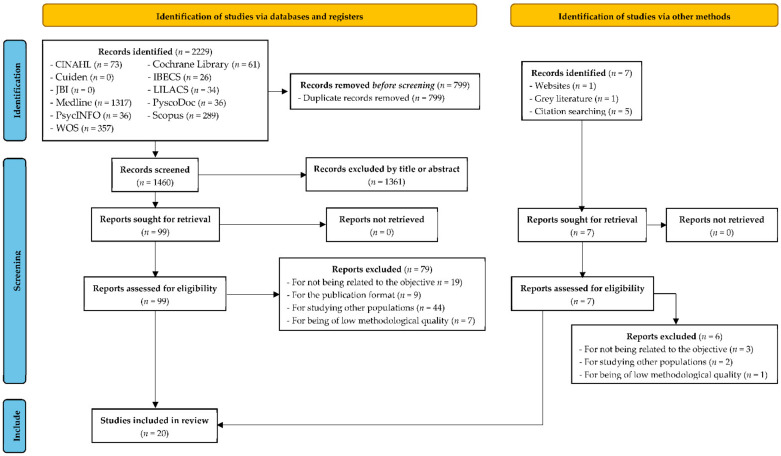
PRISMA 2020 flow diagram for the study selection process, which included searches of databases, registers, and other sources.

The initial search returned a result of 2229 identified papers, of which 799 were deleted as duplicates. After reading the title and abstract, 1361 studies were discarded because they were not aligned with the objective of the review or failed to meet the previously established criteria for inclusion. A total of seven new references were found when completing an inverse manual search. With the 99 studies that were considered potentially relevant and met the eligibility criteria, critical readings of their complete texts led to the removal of 22 that were unrelated to the objective of the review, 9 because of their publication format (editorials or opinion-based articles), 46 for contributing no data from out-of-hospital HPs, and 8 for not reaching the minimum required score in the evaluation of their methodological quality. Finally, 20 articles were agreed upon to form part of the review (Figure 3). With regard to the selection process of the studies, from among the 99 articles considered of potential relevance, each evaluator undertook a critical reading of 25, requiring the participation of a third evaluator on 7 occasions. At no time was it necessary to contact the authors of the studies.

Neither a meta-analysis nor a meta-synthesis could be conducted with the studies included in the review due to the high levels of observed heterogeneity between the participants, in the area of development, in the scales and measurement instruments used, and with the final results. Furthermore, areas of uncertainty related to the scarcity of studies carried out with EMS professionals were identified since the COVID-19 pandemic is a recent event, never before experienced, which limits the scientific literature on this phenomenon. The update plan of this systematic review will allow the identification of new studies about stress, anxiety, depression, and self-efficacy in out-of-patients HPs. A meta-analysis or a meta-synthesis will be performed to analyze the evidence of any new eligible studies or data that are obtained.

A narrative synthesis of the main characteristics and findings from those studies was performed, which is summarized in Table 2.

### 3.1. Description of the Characteristics of the Studies

The designs of most of the selected research (*n* = 16) were of the descriptive cross-sectional type [52,54,55,56,57,59,60,61,62,63,64,65,66,67,68,69], except in the case of the 4 remaining studies in which a qualitative methodology was used [51,53,58,70]. With regard to their geographic distribution, the studies were completed in regions and countries with important differences in the organization of their health systems: Italy (*n* = 6) [56,63,64,65,66,67], Germany (*n* = 2) [52,59], Spain (*n* = 2) [57,61], Turkey (*n* = 2) [55,62], Belgium (*n* = 1) [68], India (*n* = 1) [53], Iran (*n* = 1) [51], Pakistan (*n* = 1) [58], Poland (*n* = 1) [54], Russia (*n* = 1) [60], and the United States (*n* = 1) [69]. It is worth mentioning that professionals from four different countries (Canada, Ireland, Kenya and the United States) participated in one of the qualitative studies [70].

The levels of stress, anxiety, depression, and self-efficacy of the out-of-hospital HPs during the COVID-19 pandemic were some of the results analyzed in the selected studies. Other results such as fear, sleep quality, resilience level, and substance abuse were not objects of evaluation in the present review, despite having been recurrent topics in the works under analysis. In most studies, the area of work was considered yet another secondary variable, including all the HPs regardless of the area in which they might work [54,55,56,59,60,61,63,64,65,66,67,68]. No great differences were found in the inclusion and exclusion criteria under consideration. In the majority of works, the participants had to be practicing HPs who, during the COVID-19 pandemic, were working in a health institution and/or organization. A high degree of variability was observed with regard to the number and professional category of the out-of-hospital health workers in the included and analyzed studies. The sample size ranged from 31 to 1831 participants in the descriptive, cross-sectional studies [55,68], while in the qualitative-based studies, this interval was between 3 and 31 participants [53,70]. Paramedics and emergency ambulance technicians were the two professional categories with the highest number of representatives in 11 of the 20 selected articles. Participants´ profession was not specified in some of the studies under review, in which they were given generic headings such as EMS, ambulance, and emergency workers [56,61,63,64,65,67,69]. Some authors considered collectives that could act as first-aid responders, such as firefighters, police, Civil Protection staff, and Red Cross volunteers, and as out-of-hospital emergency personnel, although they were not HPs [56,63,65,66,69].

In the descriptive, cross-sectional studies, the participants were selected through non-probabilistic convenience sampling based on voluntary participation. In all of them, except in one, data collection was done through the completion of an online survey distributed through the principal social networks (Facebook^®^, Instagram^®^, LinkedIn^®^, Twitter^®^, WhatsApp^®^), email lists, and specialized fora and web pages of public organizations and thematic communities [52,54,55,56,57,59,60,61,63,64,65,66,67,68,69]. Among all the quantitative investigations, the study of Usul et al. was the only one that decided to conduct personal interviews with each of the participants as a data-collection method [62]. The necessary time to complete the survey fluctuated between 5 and 20 min. The period analyzed in most studies included in this review was the first phase of the COVID-19 pandemic, between the months of March and July 2020, which coincided with an important increase in the global case rate as well as with the adoption of restrictive governmental measures in the great majority of countries, which considerably constrained the individual liberties of the general public [52,54,55,56,57,59,60,61,66,69]. The study by Vagni et al. was the only one in which data was collected on both waves of the pandemic [67]. Different questionnaires and scales, of which most were validated, were used to evaluate the possible impact of exposure to SARS-CoV-2 on the mental health of out-of-hospital HPs. Stress levels were assessed through the Emergency Stress Questionnaire (ESQ) [56,63,64,65,66,67], the Perceived Stress Scale (PSS) [67], the Psychological Stress Measure (PSM-25) [60], and ad hoc questionnaires on stressful factors [52,54,56,63,64]. The Secondary Traumatic Stress Scale—Italian version (STSS-I) [56,63,65,66,67], the Davidson Trauma Scale (DTS-8) [57], and the Post-traumatic Stress Disorder Checklist for DSM-5 adapted to COVID-19 (PCL-5) [69] were the questionnaires used to evaluate the presence of post-traumatic stress. The development of burnout was valued through the use of the Maslach Burnout Inventory [61,64,67]. The State-Trait Anxiety Inventory (STAI) [55,62], the Generalized Anxiety Disorder 7-items (GAD-7) [59], the Generalized Anxiety Disorder 2-items (GAD-2) [52], and the Overall Anxiety Severity and Impairment Scale (OASIS) [69] were chosen to quantify the level of anxiety. The presence of depressive symptoms was evaluated through the Patient Health Questionnaire 2 (PHQ-2) [52,59] and the Overall Depression Severity and Impairment Scale (ODSIS) [69]. The Coping Self-Efficacy Scale—Short Form (CSES-SF) was the tool chosen to value self-efficacy [56,63,64,65,66,67]. Other questionnaires or ad hoc lists were used, such as Attitude of Healthcare Workers towards COVID-19 Pandemic [61], General Health Questionnaire-12 (CHQ-12) [57], Frequency of Negative and Positive Mental Health Symptoms [68], and the Mental Health Correlates Questionnaire (MHCQ) [69], in which the state of mental health of HPs was evaluated through the presence of certain symptoms. Taking into account the characteristics and instructions of each one of these instruments, the collected data could refer to the time at which the questionnaires were completed, the week immediately before, or other points in time. With regard to the statistical analysis, in most studies, univariate tests were employed to analyze the effect of the different variables on the principal result of the study. In some of them, it was also complemented with multivariate tests for the simultaneous analysis of various variables and the identification of possible predictive factors [52,54,56,61,63,64,65,66,67,68,69].

An intentional non-probabilistic sampling was used in the investigations that followed a qualitative methodology, with the purpose of achieving the greatest possible variability in the selection of the participants. Munawar et al. and Zolkinov et al. opted to use social networks during the participant recruitment process [58,70]. The time period for data collection was between March and July 2020 [51,58]. A semi-structured in-depth interview was used as a data-collection instrument, conducted by researchers with expertise in this type of study via telephone [51], video calls [51,70], or in person [53,58]. George et al. complemented this interview with the creation of four focal discussion groups in which there were 8-to-11 participants [53]. Thematic analysis was used for data interpretation in two studies [51,58]; in another, ethnographic analysis with an interpretative focus was used [53], and, in yet another, phenomenological descriptive analysis [70].

### 3.2. Description of the Results of the Critical Evaluation of the Studies

The results of the critical appraisal of the 16 selected studies are summarized in Table 3 and Table 4. The majority of the studies scored moderately due to the unclear description of certain details of the methodology used. In the descriptive, cross-sectional studies, these details referred to the form of evaluating the mental health of HPs as well as to the way of identifying and controlling possibly confounding factors; in the qualitative studies, these details referred to the possible influence of the researcher in the study and compliance with the minimum necessary ethical requirements. Only one qualitative study adequately described all the details required according to the critical appraisal applied [70].

### 3.3. Description of the Results of the Studies

#### 3.3.1. Stress

In the selected studies, the level of stress of the HPs was assessed from different perspectives: acute stress, post-traumatic stress, and burnout.

##### Acute Stress

From among the 20 selected articles, 14 specifically evaluated stress levels [51,52,53,54,56,58,60,63,64,65,66,67]. The 4698 out-of-hospital participants included EMS workers, ambulance workers, paramedical personnel, firefighters, police, Civil Protection staff, and Red Cross volunteers. The total stress levels did not differ between the two waves of the pandemic [67]. In relation to the previous situation, a greater frequency of stress was observed among the paramedics during the COVID-19 pandemic [68]. In comparison with other HPs, the out-of-hospital workers obtained lower levels of total stress, organizational–relational stress, physical stress, inefficacy–decisional stress, emotional stress, cognitive stress, and COVID-19 stress. Women experienced physical and emotional stress with greater frequency [65,66], while the men scored significantly higher for inefficacy–decisional stress [65]. Older-aged workers obtained higher levels of organizational–relational stress, physical stress, emotional stress, and cognitive stress [64,65,66]. Nurses experienced higher levels of stress at work than paramedics during the pandemic [54]. The hours of weekly work had no effect on the subscales of stress [54,66]. Having direct contact with patients affected by COVID-19 or the fear of contracting the disease favored the appearance of total stress and inefficacy–decisional stress [54,56,64], not observing any effect of this variable in a study completed by Vagni et al. [65]. Non-availability of adequate personal protective equipment and a decrease in the level of safety and security while conducting emergency medical procedures were the risk factors for the development of organizational relational stress, physical stress, emotional stress, cognitive stress, and total stress [54,66]. Another predictor of occupational stress was the marginalization of patients not suffering from COVID-19 [54]. When asking the participants in one of the qualitative studies on possible stress-related factors, the following emerged: uncertainty over the pandemic, fear of death, the feeling of guilt for having passed the illness to their loved ones, anxiety over the likelihood of violence from patients, and exhaustion [53].

##### Post-Traumatic Stress

With regard to post-traumatic stress, this aspect is specifically analyzed in seven articles [56,57,63,65,66,69]. The 1485 out-of-hospital HPs who participated in the studies were distributed among EMS workers, ambulance workers, firefighters, firefighters-EMS law enforcement, police, Civil Protection staff, and Red Cross volunteers. 6.8% of participants presented symptoms consistent with post-traumatic stress [69]. The degree of physiological and psychological activation was altered with greater frequency among both women and out-of-hospital HPs [56,65,66,69]. Being a woman and being an older person were related to higher levels of avoidance behavior and obsessive thoughts [66]. The weekly hours of work had no effect on the symptoms of secondary trauma [66]. The risk of probability of developing symptoms of post-traumatic stress was related to gender, changes in job functions, having had prior theoretical and practical training of the use of personal protective equipment, having been worried about contracting the disease, anxiety symptoms prior to and during the pandemic, use of anxiolytics during the pandemic, requiring psychological support prior and during the pandemic, and dealing with mental health issues normally in the work unit [57]. The non-availability of adequate personal protective equipment was a risk factor for the development of post-traumatic stress and avoidance behavior [57,66]. Greater concern over COVID-19 was related to a higher probability of developing symptoms of post-traumatic stress [69].

##### Burnout

The level of burnout was evaluated in 3 of the 20 selected studies [61,64,67], in which the sample was formed of 309 EMS workers and 157 volunteers. In all the subscales of stress, a positive correlation was observed with emotional exhaustion and depersonalization and a negative correlation was observed with personal accomplishment [64]. In the second wave of the pandemic, total stress levels showed a high predictive power in emotional exhaustion and depersonalization [67]. Torrente et al. observed no differences in levels of burnout among HPs as a function of professional category and field of work [61]. In turn, Vagni et al. demonstrated that personal accomplishment was reduced among out-of-hospital workers of older age, increasing among those entering into direct contact with COVID-19 patients [64].

#### 3.3.2. Anxiety

Anxiety levels were evaluated in 8 of the 20 studies [51,52,55,58,59,62,69,70]; 2133 individuals participated, of whom 20 were physicians, 14 were nurses, 1590 were paramedics, 251 were emergency medical technicians, 53 were drivers, 43 were EMS-workers, 63 were firefighters, 93 were firefighters—EMS, 3 were firefighters—EMS law enforcement, and 3 were police. Around 15–20% of the participants met the established clinical criteria for anxiety [54,69]. The levels of anxiety reported by women were greater than those of men, observing a reduction as the age of the participants increased [62]. With regard to professional category and scope of work, the results from the different studies were not conclusive: Karasu et al. observed no differences in anxiety levels [55]; Skoda et al. affirmed that paramedics obtained the lowest levels of anxiety [59], while for Usul et al. lowest levels of anxiety were experienced by nurses [62]. Having personal antecedents of any medical condition that increased the risk of suffering COVID-19, being concerned over infecting family and/or friends, thinking they had no proper personal protective equipment, or feeling more nervous were factors that significantly increased the anxiety levels of out-of-hospital HPs [62,69]. HPs with symptoms of anxiety were more frequently reported to be burdened by an increase in workload due to the pandemic, thoughts about SARS-CoV-2 contraction at the workplace, a shortfall of colleagues, the childcare situation, not being able to let patients down, uncertainty about how to act correctly, uncertainty about contact persons, uncertainty about their financial situation, and uncertainty about temporal scope [52]. Other factors identified in the qualitative studies were the high workloads, the feeling of having lost control of the situation, the feeling of not being useful, isolation and separation from loved ones, lack of support and understanding among family members and friends, and the fear of dying [51,70]. Treating patients with COVID-19 or suspected cases of COVID-19 was a factor that induced anxiety in the study carried out by Usul et al. [62], observing no influence at all in the one by Vujanovic et al. [69]. Moreover, thinking that sufficient and adequate information on COVID-19 was available was the only factor that was related to a reduction in anxiety levels [59].

#### 3.3.3. Depression

The presence of depressive symptoms was evaluated in 3 of the 20 selected articles [52,59,69]. The sample of out-of-hospital health workers comprised 333 women, 1610 men and 2 diverse individuals, distributed among 1499 paramedics, 35 EMS workers, 60 firefighters, 91 firefighters—EMS, and 3 firefighters—EMS law enforcement. Around 15% of the participants met with the clinical criteria established for a medical history of depression [69]. No statistically significant differences were observed in the levels of depression of the participants as a function of their professional category [59]. The out-of-hospital HPs at greater risk of presenting depressive-type symptoms were those who expressed concern over the COVID-19 pandemic, those suffering any illness that increased the risk of being infected with COVID-19, those who did not feel protected by personal protective equipment, and those with less work experience [52,69]. Direct assistance to patients showing signs of COVID-19 or with confirmed cases was unrelated to the frequency of depressive symptoms [69]. The stressors that increased the likelihood of suffering depressive symptoms were: a shortfall of colleagues, not being able to let patients down, uncertainty about how to act correctly, uncertainty about contact persons, uncertainty about their financial situation, and uncertainty about temporal scope [52].

#### 3.3.4. Self-Efficacy

The self-efficacy of out-of-hospital professionals was evaluated in 6 of the 20 selected articles [53,56,58,63,66,67]. The sample was formed of 892 participants, in which the professional categories of EMS workers, firefighters, Civil Protection staff, and voluntary Red Cross personnel were analyzed. Out-of-hospital workers used multiple adaptative interventions and coping strategies in order to promote perceptions of self-efficacy. Some were centered on the thoughts and emotions of the person, with the aim of favoring cognitive reappraisal and positive reframing; others were focused on the problem in order to reduce the risk of infection, and yet others on the meaning of the situation, so that the stressful experience could help to maintain personal wellbeing in difficult times [53]. Among them, “stop unpleasant emotions and thoughts” was the strategy used with greater frequency by the out-of-hospital workers [53,63], acting as a predictive factor of less organizational–relational stress, physical stress, emotional stress, cognitive stress, and COVID-19 stress as well as a lesser degree of physiological and psychological activation and obsessive thoughts as symptoms of secondary trauma [66]. In turn, the men resorted to problem-focused strategies to confront the different situations that might present themselves during the COVID-19 pandemic [66]. Use of the coping strategy of support was predictive of fewer instances of avoidance behavior as a symptom of secondary trauma [66]. No differences were observed in the coping strategies used by the participants between the two waves of the pandemic [67]. In the qualitative study developed by Munawar et al., the 15 emergency ambulance technicians/drivers who were interviewed affirmed that they turned with greater frequency to the following coping strategies to face the situation of uncertainty derived from the COVID-19 pandemic: religion, passion for serving their community and country, the feeling of having complied with their commitment, altruism, empathy, non-exposure to the communications media, and thinking that it is just another emergency [58].

## 4. Discussion

In this living systematic review, the impact of the COVID-19 pandemic on the mental health of out-of-hospital HPs has been evaluated. Their results have revealed the existence of different factors that have been associated with a greater or lesser risk of developing symptoms of stress, anxiety, and depression as well as the identification of various coping strategies used by HPs to adapt to the situations arising from the COVID-19 pandemic.

Being a woman was associated with higher levels of perceived anxiety and stress, similar results to those obtained by health workers from other areas [16,71,72,73,74,75,76]. Likewise, the women recognized that they had had a high degree of physiological and emotional activation, avoidance behaviors, and obsessive thoughts, with greater frequency than men, which are factors that are related to greater susceptibility to the development of post-traumatic stress [76,77]. These results may be due to a series of elements that favor an effect on their mental wellbeing, such as the high feminization of the health sector, discrimination by gender, the difficulty of conciliating family life with work life, the consequences associated with pregnancy and maternity, the lack of support systems, the greater empathetic capacity of women in providing care and helping others, and their greater ability to express their feelings and to develop emotional responses in the face of stressful events [76,78,79,80]. These factors considerably reduce the time that may be dedicated to self-care and self-compassion, which favors the maintenance of this situation over time [80,81]. In addition, the women tended to employ emotion-focused coping strategies, which are less effective under stressful or emergency situations [82,83]. Problem-focused strategies were mainly used by men, limiting their capacity to recognize their emotional difficulties and to create awareness of their own experiences. This strategy is related to a higher risk of presenting psychosomatic complications as a consequence of perceived stress [83].

Age had no uniform influence on the mental health of out-of-hospital workers, in agreement with the results obtained from other studies [84,85,86,87,88,89,90]. On the one hand, the older-aged professionals were more vulnerable to the development of symptoms of stress during the COVID-19 pandemic due to the high pressure they were under while at work, their feelings of responsibility towards their colleagues and society, and a greater awareness of the risks and seriousness of the situation [84]. The feeling of having lost control of the situation and the fear of infection were lived as a continual threat against their own health and that of their family members, especially in the first phases, due to the lack of existing information and knowledge [84,85]. In addition, an older age was also associated with the adoption of avoidance strategies towards negative thoughts and emotions, which means that the person may be less influenced by intrusive components of their previous traumatic experiences [86]. On the other hand, their levels of anxiety progressively diminished as their age increased, converting it into a protective factor, which could be related to a higher level of competence and experience at work, a greater feeling of self-esteem, and the activation of internal resources and personal skills for handling adverse situations [89,90,91]. This protective effect of age was also observed in the development of symptoms of secondary trauma, especially in the studies that were performed after the initial phase of the pandemic [66].

Having a personal history of any pathology that increased susceptibility to COVID-19 infection was related to higher levels of anxiety and depression, which may be due to a greater concern about infection and its consequences as it implies a higher risk in this group of people. In other areas, similar results have been observed for the presence of depressive symptoms [92] but not for the anxious type [93,94]. Among HPs from other areas, having a personal history of any mental health disorder, especially those related to substance abuse or depression, was strongly associated with the presentation of a current mental disorder. The higher the number of prior lifetime mental disorders reported, the more likely the prevalence of any current disorder [95].

With regard to professional category and field of work, the results from the studies under analysis were neither unanimous nor conclusive, which can be related to the heterogeneity in the organizations and functional operations of the health systems and out-of-hospital EMS within the different countries. Contrary to what might be expected and to what has been reported in other studies [24,96], Usul et al. were the only authors who identified out-of-hospital nursing professionals as presenting lower levels of anxiety [62]. In various studies, the impact of the COVID-19 pandemic on the mental health of out-of-hospital health workers and volunteers has been analyzed, comparing their results with those obtained by HPs from other areas. In all of them, levels of anxiety and stress were high, which exposed them to a greater risk of developing symptoms of secondary trauma; however, these levels were lower than those of out-of-hospital workers, especially among paramedics. These challenges can be related to the fact that the front-line workers perceived their actions as the continuity of their habitual procedures and activities, although with higher levels of self-protection and safety, as they were more accustomed to potentially worrying experiences and showed fewer negative responses in the face of challenging situations [44,97,98]. In the case of the volunteers, personal motivation, freedom to choose weekly hours of service dedicated to voluntary work, and feelings of gratification when offering their support to others might be the cause of their low stress levels, although they have a greater risk of suffering emotional exhaustion, as happens with the caring professions [16,99,100].

No significant differences have been observed in the impact of the COVID-19 pandemic on the mental health of out-of-hospital HPs in different regions. However, this situation has shown the existence of important deficiencies in the health systems of the countries, regardless of their level of economic development. In many low- and middle-income countries, the consequences of the pandemic on front-line health workers have been particularly severe due to limited resources, insufficiently funded health facilities and understaffing, a lack of skills such as self-resilience in HPs, and a lack of support from government agencies and institutions. Many of these consequences have also been observed in higher-income countries [101].

Specific factors of this pandemic have been perceptions of insecurity and fear of getting infected or infecting family members, which have directly impacted the mental health of HPs and their professional performance [85,102]. The out-of-hospital HPs who entered into direct contact with patients showing signs of COVID-19 or confirmed cases presented greater risks of developing high levels of anxiety and stress [16], a situation that might have been aggravated due to the lack of personal protective equipment, the non-existence of clear and specific action protocols, difficulties with the reorganization of health systems, and limited knowledge of the illness, especially in the first stage of the pandemic [24,57,98,103]. In successive phases, when action protocols were finally available, there was a greater knowledge of the illness, and the number of people affected was significantly lower; the HPs had more time for retrospective reflection on their actions and interventions, a fact that was associated with feelings of guilt, frustration and regret, and a feeling of inefficacy [26,104,105]. The weekly working hours had no effect on levels of anxiety and stress nor the somatic manifestations of secondary trauma. The reactions of the HPs were not, therefore, related to the time dedicated to attention to the patient but to how they perceived the situation. The lack of both effective personal protective equipment and specific and adequate instructions and knowledge increased the risk of infection, affecting proper decision-making among HPs, increasing their feeling of deficient and ineffective control, and favoring the emergence of conflicts in the organization and in relation to colleagues [89,97,98,103]. With a coping strategy based on stopping unpleasant thoughts and emotions, these workers limited their feeling of impotence and incapability, favoring the activation of proactive attitudes, attenuating nervous reactions, and reducing levels of anxiety and stress [26]. Therefore, the impact of these variables was contained and limited by the use of adaptative strategies and coping strategies [96].

The adaptative strategies to which the out-of-hospital HPs resorted most frequently to reduce the impact of the COVID-19 pandemic on their mental health, in a general manner, were to stop unpleasant emotions and thoughts and positive reframing. These professionals had to give rapid and immediate responses in high-pressure situations in which they were living. It meant that they concentrated on what they had to do, with no time to think and to reflect on their own emotions, and they thereby promoted and reinforced positive and optimistic attitudes and avoided pain, impotence, and guilt [26,106]. In this way, processes such as pondering, reflection, and emotional and cognitive self-awareness were inhibited, whereas other more objective aspects such as the lack of personal protective equipment or clear and specific action protocols had a direct impact on the mental health of these HPs [106]. It was not seen that the problem-focused strategy had a protective effect on mental state during the first phase of the COVID-19 pandemic, which might be due to them not fully understanding the emergency against which they were fighting or not having sufficient scientific knowledge on the effective operating and therapeutic procedures to be used [89]. In addition, the absence of adequate personal protective equipment led them to perceive low self-efficacy, which was reflected in lower confidence in their capability to organize and make effective decisions. Likewise, perceiving that there was organizational support improved their self-efficacy and promoted the adoption of this type of coping strategy [107]. The coping strategy of support had a marginal effect on the mental health of the HPs due to the government of the different countries ordering lockdowns at home and social distancing measures. Requesting help in the workplace might generate situations of tension, frustration, and deception; the majority of workers found themselves in situations of physical and emotional overload [108].

In emergency situations such as the COVID-19 pandemic, it is fundamental that the planning of measures for psychological support, training, and supervision be directed at all HPs, interventions that help them to reflect on their own psycho-emotional reactions to adverse events [108,109]. The use of psychiatric teleconsultation, social support networks, and the establishment of online support groups have proven to be effective strategies for people to stay connected during the pandemic [110]. Therefore, the implementation of these measures should be incentivized by the organizations themselves, in which the workers learn to manage their levels of stress and anxiety, reduce exhaustion, increase their resilience, improve their feelings of self-efficacy and self-confidence, and strengthen their cognitive and emotional skills [109,111]. With the aim of being able to optimize their effectiveness, these interventions must be based on a multidisciplinary and individualized approach to the person, in which the variables that increase their vulnerability are taken into account.

The findings of this review must be considered in the context of their own strengths and limitations. Its principal strength might be that no other systematic review has, to date, specifically analyzed the impact of the COVID-19 pandemic on the mental health of out-of-hospital HPs. As more evident limitations of this study, arising from its own methodological approach, publication bias and selection bias in the choice of the databases consulted may be mentioned, as well as the search strategy that was used and the exclusion of works published in languages other than those previously stated. An attempt was made to minimize these sorts of bias through the participation of at least 2 researchers in the search and selection process in 11 of the most relevant databases in the field of health sciences and psychology as well as the use of a somewhat unrestrictive search strategy. The methodological quality of studies selected was optimal, except in some cases, with reference to participation rates and sample representativeness, justified by the epidemiological context and the generalized difficulties imposed as a consequence of the outbreak on both HPs and the researchers themselves. The high heterogeneity observed in the socio-demographic characteristics of the sample of participants, in the field under development and in the measurement scales and instruments that were in use, complicated the comparison and any extrapolation of the results as well as the analysis of biases. Moreover, along with the relative scarcity of published studies, it must be added that a joint and not always a comparative approach was used for data analysis in most of them, without discriminating between out-of-hospital professionals and other health personnel.

This living systematic review was conducted at a relatively early stage of the COVID-19 pandemic. In the coming months and years, many primary studies and systematic reviews will continue to be published, so the future updating of this review and the completion of a systematic review of reviews could be a new source of evidence on the topic. In addition, there is a clear need to continue investigating the life experiences and specific needs of out-of-hospital health staff during the COVID-19 pandemic and to explore the medium- and long-term consequences on their physical, mental, emotional, and social health using different designs based on qualitative methods to do so.

## 5. Conclusions

In the exercise of their functions, the mental health of out-of-hospital HPs was strained during the COVID-19 pandemic. The HPs of the female sex, those entering into direct contact with patients showing signs of COVID-19 or with confirmed cases, and others with certain personal backgrounds were more vulnerable to the development of stress, anxiety, and/or depression. The impact of the pandemic on the mental health of HPs was lower among those working in out-of-hospital EMS in relation to other areas of hospital assistance. Stopping unpleasant emotions and thoughts was the most frequently used strategy, with good results among these HPs. Planning measures for psychological support, training, and supervision is fundamental, in which help can be offered to the HPs for reflection on their psycho-emotional reactions in the face of adverse events. Possible future research should be oriented to explore the life experiences and specific needs of out-of-hospital health staff during the COVID-19 pandemic, as well as to analyze the medium- and long-term consequences on their physical, mental, emotional, and social health.

## Figures and Tables

**Table 1:** Search strategy adapted to each of the databases.

Database	Search Strategy
CINHAL	(MH “prehospital emergency care” OR “emergency care” OR “emergency system” OR “out of hospital” OR “emergency medical service*”) AND (MH “health care provider” OR “healthcare worker*” OR “health care professional*” OR “health personnel” OR “physician*, primary care” OR doctor* OR physician* OR “medical staff” OR “nursing personnel” OR “registered nurse*” OR “assistant nurse*” OR nurs* OR “nursing staff” OR “emergency paramedic*” OR paramedic* OR “paramedical personnel” OR “healthcare assistant*” OR “healthcare support worker*” OR “emergency medical technician*” OR “allied health personnel”) AND (MH “2019-nCoV infection” OR “2019-nCoV disease” OR “coronavirus disease-19” OR “severe acute respiratory syndrome coronavirus 2” OR “SARS-CoV-2 virus” OR “SARS-CoV-2 infection” OR “COVID-19” OR “SARS-CoV-2”) AND (MH “COVID-19 pandemic” OR pandemic*) AND (MH “mental health” OR angst OR nervousness OR hypervigilance OR anxiousness OR anxiety OR “anxiety disorder*” OR “depressive syndrome” OR melancholia OR “depressive symptom” OR “emotional depression” OR dysthymia OR depressi* OR “depressive disorder*” OR “dysthymic disorder*” OR “acute stress disorder*” OR “life stress” OR “psychologic stress” OR “psychological stressor” OR “stress disorders, traumatic, acute” OR “stress, psychological” OR “self-confidence” OR “self-efficacy")
Cochrane Library	“emergency medical service*” AND (“health personnel” OR physician* OR “medical staff” OR nurse* OR “nursing staff” OR “emergency medical technician*” OR “allied health personnel”) AND (“COVID-19” OR “SARS-CoV-2”) AND pandemic* AND (“mental health” OR anxiety OR “anxiety disorder*” OR depression OR “depressive disorder*” OR “stress disorder*, traumatic, acute” OR “stress, psychological” OR “self-efficacy”)
Cuiden	“servicio* medico* de urgencia” AND (“personal de salud” OR medico* OR “cuerpo médico” OR “enfermera* y enfermero*” OR “personal de enfermería” OR “técnico* medio* en salud”) AND (“infección* por coronavirus” OR “virus del SARS”) AND pandemia* AND (“salud mental” OR ansiedad OR depresión OR “trastorno* de estrés traumático agudo” OR “estrés psicológico” OR autoeficacia) [“emergency medical service” AND (“health personnel” OR doctor OR “medical body” OR “female nurse and male nurse” OR “nursing personnel” OR “health auxiliary”) AND (“infection by coronavirus” OR “SARS virus”) AND pandemic AND (“mental health” OR anxiety OR depression OR “traumatic acute stress disorder” OR “psychological stress” OR self-efficacy)]
IBECS	(tw:((“emergency medical service*” OR “servicio* medico* de urgencia”) AND (“health personnel” OR “personal de salud” OR physician* OR “medical staff” OR medico* OR “cuerpo medico” OR nurse* OR “nursing staff” OR “enfermera* y enfermero*” OR “personal de enfermería” OR “emergency medical technician*” OR “allied health personnel” OR “técnico* medio* en salud”) AND (“COVID-19” OR “SARS-CoV-2” OR “infeccion* por coronavirus” OR “virus del SARS”) AND pandemic* OR pandemia*) AND (“mental health” OR “salud mental” OR anxiety OR “anxiety disorder*” OR ansiedad OR depression OR “depressive disorder*” OR depresión OR “stress disorder*, traumatic, acute” OR “stress, psychological” OR “trastorno* de estrés traumático agudo” OR “estrés psicológico” OR “self-efficacy” OR autoeficacia)]
JBI	((prehospital emergency care OR emergency care OR emergency system OR out of hospital OR emergency medical service*) AND (health care provider OR healthcare worker* OR health care professional* OR health personnel OR physician*, primary care OR doctor* OR physician* OR medical staff OR nursing personnel OR registered nurse* OR assistant nurse* OR nurs* OR nursing staff OR emergency paramedic* OR paramedic* OR paramedical personnel OR healthcare assistant* OR healthcare support worker* OR emergency medical technician* OR allied health personnel) AND (2019-nCoV infection OR 2019-nCoV disease OR coronavirus disease-19 OR severe acute respiratory syndrome coronavirus 2 OR SARS-CoV-2 virus OR SARS-CoV-2 infection OR COVID-19 OR SARS-CoV-2) AND (COVID-19 pandemic OR pandemic*) AND (mental health OR angst OR nervousness OR hypervigilance OR anxiousness OR anxiety OR anxiety disorder* OR depressive syndrome OR melancholia OR depressive symptom OR emotional depression OR dysthymia OR depressi* OR depressive disorder* OR dysthymic disorder* OR acute stress disorder* OR life stress OR psychologic stress OR psychological stressor OR stress disorders, traumatic, acute OR stress, psychological OR self-confidence OR self-efficacy))
LILACS	(tw:((“emergency medical service*” OR “servicio* medico* de urgencia”) AND (“health personnel” OR “personal de salud” OR physician* OR “medical staff” OR medico* OR “cuerpo medico” OR nurse* OR “nursing staff” OR “enfermera* y enfermero*” OR “personal de enfermería” OR “emergency medical technician*” OR “allied health personnel” OR “técnico* medio* en salud”) AND (“COVID-19” OR “SARS-CoV-2” OR “infeccion* por coronavirus” OR “virus del SARS”) AND pandemic* OR pandemia*) AND (“mental health” OR “salud mental” OR anxiety OR “anxiety disorder*” OR ansiedad OR depression OR “depressive disorder*” OR depresión OR “stress disorder*, traumatic, acute” OR “stress, psychological” OR “trastorno* de estrés traumático agudo” OR “estrés psicológico” OR “self-efficacy” OR autoeficacia)
Medline	(((emergency medical services[MeSH Terms] OR “prehospital emergency care”[All Fields] OR “emergency care” [All Fields] OR "emergency system"[All Fields] OR "out of hospital"[All Fields] OR “emergency medical service*” [All Fields]) AND (health personnel[MeSH Terms] OR “health care provider”[All Fields] OR “healthcare worker*”[All Fields] OR “health care professional*”[All Fields] OR “health personnel”[All Fields]) OR (physicians[MeSH Terms] OR medical staff[MeSH Terms] OR “physician*, primary care”[All Fields] OR doctor*[All Fields] OR physician*[All Fields] OR “medical staff” [All Fields]) OR (nurse[MeSH Terms] OR nursing staff[MeSH Terms] OR “nursing personnel”[All Fields] OR “registered nurse*”[All Fields] OR "assistant nurse*"[All Fields] OR nurs*[All Fields] OR “nursing staff” [All Fields]) OR (emergency medical technicians[MeSH Terms] OR allied health personnel[MeSH Terms] OR “emergency paramedic*”[All Fields] OR paramedic*[All Fields] OR “paramedical personnel”[All Fields] OR “healthcare assistant*”[All Fields] OR “healthcare support worker*”[All Fields] OR “emergency medical technician*”[All Fields] OR “allied health personnel”[All Fields])) AND ((COVID-19[MeSH Terms] OR SARS-CoV-2[MeSH Terms] OR “2019-nCoV infection”[All Fields] OR “2019-nCoV disease”[All Fields] OR “coronavirus disease-19”[All Fields] OR “severe acute respiratory syndrome coronavirus 2”[All Fields] OR “SARS-CoV-2 virus”[All Fields] OR “SARS-CoV-2 infection”[All Fields] OR “COVID-19”[All Fields] OR “SARS-CoV-2”[All Fields]) AND (pandemics[MeSH Terms] OR “COVID-19 pandemic”[All Fields] OR pandemic*[All Fields])) AND ((mental health[MeSH Terms] OR “mental health”[All Fields]) OR (anxiety[MeSH Terms] OR anxiety disorders[MeSH Terms] OR angst[All Fields] OR nervousness[All Fields] OR hypervigilance[All Fields] OR anxiousness[All Fields] OR anxiety[All Fields] OR “anxiety disorder*”[All Fields]) OR (depression[MeSH Terms] OR depressive disorder[MeSH Terms] OR “depressive syndrome”[All Fields] OR melancholia[All Fields] OR “depressive symptom*”[All Fields] OR “emotional depression”[All Fields] OR dysthymia[All Fields] OR depression[All Fields] OR “depressive disorder*”[All Fields]) OR (stress disorders, traumatic, acute[MeSH Terms] OR stress, psychological[MeSH Terms] OR “acute stress disorder”[All Fields] OR “life stress”[All Fields] OR “psychologic stress”[All Fields] OR “psychological stressor”[All Fields] OR “stress disorders, traumatic, acute”[All Fields] OR “stress, psychological”[All Fields]) OR (self-efficacy[MeSH Terms] OR "self-confidence"[All Fields] OR “self-efficacy”[All Fields])))
PyscoDoc	(prehospital emergency care OR emergency care OR emergency system OR out of hospital OR emergency medical service*) AND (health care provider OR healthcare worker* OR health care professional* OR health personnel OR physician*, primary care OR doctor* OR physician* OR medical staff OR nursing personnel OR registered nurse* OR assistant nurse* OR nurs* OR nursing staff OR emergency paramedic* OR paramedic* OR paramedical personnel OR healthcare assistant* OR healthcare support worker* OR emergency medical technician* OR allied health personnel) AND (2019-nCoV infection OR 2019-nCoV disease OR coronavirus disease-19 OR severe acute respiratory syndrome coronavirus 2 OR SARS-CoV-2 virus OR SARS-CoV-2 infection OR COVID-19 OR SARS-CoV-2) AND (COVID-19 pandemic OR pandemic*) AND (mental health OR angst OR nervousness OR hypervigilance OR anxiousness OR anxiety OR anxiety disorder* OR depressive syndrome OR melancholia OR depressive symptom OR emotional depression OR dysthymia OR depressi* OR depressive disorder* OR dysthymic disorder* OR acute stress disorder* OR life stress OR psychologic stress OR psychological stressor OR stress disorders, traumatic, acute OR stress, psychological OR self-confidence OR self-efficacy)
PsycINFO	(prehospital emergency care OR emergency care OR emergency system OR out of hospital OR emergency medical service*) AND (health care provider OR healthcare worker* OR health care professional* OR health personnel OR physician*, primary care OR doctor* OR physician* OR medical staff OR nursing personnel OR registered nurse* OR assistant nurse* OR nurs* OR nursing staff OR emergency paramedic* OR paramedic* OR paramedical personnel OR healthcare assistant* OR healthcare support worker* OR emergency medical technician* OR allied health personnel) AND (2019-nCoV infection OR 2019-nCoV disease OR coronavirus disease-19 OR severe acute respiratory syndrome coronavirus 2 OR SARS-CoV-2 virus OR SARS-CoV-2 infection OR COVID-19 OR SARS-CoV-2) AND (COVID-19 pandemic OR pandemic*) AND (mental health OR angst OR nervousness OR hypervigilance OR anxiousness OR anxiety OR anxiety disorder* OR depressive syndrome OR melancholia OR depressive symptom OR emotional depression OR dysthymia OR depressi* OR depressive disorder* OR dysthymic disorder* OR acute stress disorder* OR life stress OR psychologic stress OR psychological stressor OR stress disorders, traumatic, acute OR stress, psychological OR self-confidence OR self-efficacy)
Scopus	(ALL(“prehospital emergency care” OR “emergency care” OR “emergency system” OR “out of hospital” OR “emergency medical service*”)) AND (ALL(“health-care provider” OR “health-care worker*” OR “health care professional*” OR “health personnel” OR “physician*, primary care” OR doctor* OR physician* OR “medical staff” OR “nursing personnel” OR “registered nurse*” OR “assistant nurse*” OR nurs* OR “nursing staff” OR “emergency paramedic*” OR paramedic* OR “paramedical personnel” OR “healthcare assistant*” OR “healthcare support worker*” OR “emergency medical technician*” OR “allied health personnel”)) AND (ALL(“2019-nCoV infection” OR “2019-nCoV disease” OR “coronavirus disease-19” OR “severe acute respiratory syndrome coronavirus 2” OR “SARS-CoV-2 virus” OR “SARS-CoV-2 infection” OR “COVID-19” OR “SARS-CoV-2”)) AND (ALL(“COVID-19 pandemic” OR pandemic*)) AND (ALL(“mental health” OR angst OR nervousness OR hypervigilance OR anxiousness OR anxiety OR “anxiety disorder*” OR “depressive syndrome” OR melancholia OR “depressive symptom” OR “emotional depression” OR dysthymia OR depressi* OR “depressive disorder*” OR “dysthymic disorder*” OR “acute stress disorder*” OR “life stress” OR “psychologic stress” OR “psychological stressor” OR “stress disorders, traumatic, acute” OR “stress, psychological” OR “self-confidence” OR “self-efficacy”))
World of Science (WoS)	(TS = (“prehospital emergency care” OR “emergency care” OR “emergency system” OR “out of hospital” OR “emergency medical service*”)) AND (TS = (“health-care provider” OR “healthcare worker*” OR “health-care professional*” OR “health personnel” OR “physician*, primary care” OR doctor* OR physician* OR “medical staff” OR “nursing personnel” OR “registered nurse*” OR “assistant nurse*” OR nurs* OR “nursing staff” OR “emergency paramedic*” OR paramedic* OR “paramedical personnel” OR “healthcare assistant*” OR “healthcare support worker*” OR “emergency medical technician*” OR “allied health personnel”)) AND (TS = (“2019-nCoV infection” OR “2019-nCoV disease” OR “coronavirus disease-19” OR “severe acute respiratory syndrome coronavirus 2” OR “SARS-CoV-2 virus” OR “SARS-CoV-2 infection” OR “COVID-19” OR “SARS-CoV-2”)) AND (TS = (“COVID-19 pandemic" OR pandemic*)) AND (TS = (“mental health” OR angst OR nervousness OR hypervigilance OR anxiousness OR anxiety OR “anxiety disorder*” OR “depressive syndrome” OR melancholia OR “depressive symptom” OR “emotional depression” OR dysthymia OR depressi* OR “depressive disorder*” OR “dysthymic disorder*” OR “acute stress disorder*” OR “life stress” OR “psychologic stress” OR “psychological stressor” OR “stress disorders, traumatic, acute” OR “stress, psychological” OR “self-confidence” OR "self-efficacy”))

The * is used to search for terms with the same root.

**Table 2:** Characteristics of the studies included in the living systematic review.

Study/Author	Objetive/Methodology	Main Findings
Arbedilli et al. [51] 2021	Objective: To explore in-depth experiences and the mental health consequences for health professionals (HPs) working due to the COVID-19 crisis. Design: Qualitative with thematic analysis. Participants: HPs who either worked directly or indirectly with cases of COVID-19. Setting: Tehran, Qom and Rasht (Iran). Procedure—Data collection: Semi-structured in-depth interviews, carried out by 4 interviewers (1 female, 3 males), university doctors, experts in qualitative studies, between 10 March and 4 July 2020, via telephone or video call. Maximum variation sampling was used to try to gather the experience of all professional categories.	*n* = 86 participants (42 females, 44 males; aged 35.34 ± 6.90 years; work experience 10.04 ± 6.08 years; 36 nurses, 17 managers, 19 physicians, 8 EMS personnel, 2 pharmacists, 2 radiologists, and 2 lab technicians) and 97 in-depth interviews (duration 34–61 min). The main topics and subtopics that emerged from the data analysis were: 1—Working in the pandemic era (overwhelming workload, ambiguity, losing control over the situation, shortage of protective devices and the difficulty of using them, feeling of futility providing care, sense of conscientiousness and self-sacrifice); 2—Changes in personal life and enhanced negative effect (fundamental changes in daily life, self-quarantining, fear of transmitting the disease to family members, fear of dying alone and being separated from loved ones, feelings of guilt and remorse); 3—Gaining experience, normalization, and adaptation to the pandemic (gaining experience, regaining self-confidence, normalization of life, adaptation to the pandemic, worries about the future, giving up protection measures); and 4—Mental health considerations (stage-wise approach, individual-centered considerations). A 3-level model was designed (early exposure, peak of crisis, and long-term effects) in which the emotions and psychological effects were identified. A high level of stress, fear, and anxiety was observed among the HPs. The sensation of impotence, despair, and abandonment prevailed among them. Many expressed fears over having lost control of the situation and that their previous knowledge and skills could not help them in this crisis. The majority were concerned about their own and their families’ state of health, but, despite anything else, they continued working, seeing themselves obliged to remain in self-confinement or to be distanced from their family members for lengthy periods of time. The provision of psychological help to HPs should be individualized, centering on the different levels obtained, in order to face up to the pandemic over lengthy periods.
Dreher et al. [52] 2021	Objective: To investigate attitudes and stressors related to the COVID-19 outbreak among EMS workers in Germany. Design: Descriptive cross-sectional. Participants: EMS workers over 18 years old. Setting: Germany. Data collection: Online survey distributed through social media channels from 9–30 April and again from 14–21 May 2020. Main outcomes—Instruments: Acute stress—ad-hoc questionnaire on stressful factors; Anxiety—GAD-2; Depression—PHQ-2.	*n* = 1278 EMS paramedics (257 females, 1278 males; median aged 32 years (interquartile range, 28–37); 19 had been previously infected by SARS-CoV-2; 1407 reported having a good or very good self-rated health). Uncertainty about the temporal scope of the pandemic and one´s childcare situation were identified as the main stressors; 16.1% of EMS workers screened positive for symptoms of anxiety, whereas 15.3% had depressive symptoms. Men were less likely to feel uncertain about correct behavior (OR 0.56; 95%CI 0.39–0.79) and less likely to feel burdened by their childcare situation (OR 0.37; 95%CI 0.14–0.95). Thoughts about SARS-CoV-2 contraction at the workplace were less common among EMS workers with higher education than among those with lower education (OR 0.37; 95%CI 0.20–0.69). Increased likelihood of feeling burdened by a shortfall of colleagues was found for older age EMS professionals (OR 1.74; 95%CI 1.27–2.49) and those with suspected or confirmed cases among colleagues (OR 1.84; 95% 1.41–3.39). Having suspected or confirmed cases among their friends or family members increased the uncertainty about contact persons (OR 1.68; 95%CI 1.23–2.31). HPs with children under care in the same household were more likely to feel uncertain about how to act correctly (OR 1.60; 95%CI 1.17–2.19), uncertain about contact persons (OR 1.54; 95%CI 1.13–2.10), and uncertain about the temporal scope of the pandemic (OR 1.73; 95%CI 1.16–2.60). EMS workers with symptoms of anxiety were significantly more frequently reported to be burdened by an increase in workload due to pandemic (OR 2.46; 95%CI 1.71–3.43), thoughts about SARS-CoV-2 contraction at the workplace (OR 4.00; 95%CI 2.60–6.16), shortfall of colleagues (OR 2.62; 95%CI 1.81–3.79), childcare situation (OR 2.34; 95%CI 1.09–5.00), not being able to let patients down (OR 1.95; 95%CI 1.35–2.82), uncertainty about how to act correctly (OR 2.50; 95%CI 1.66–3.76), uncertainty about contact persons (OR 1.67; 95%CI 1.6–2.40), uncertainty about their financial situation (OR 2.65; 95%CI 1.77–3.98), and uncertainty about temporal scope (OR 2.79; 95%CI 1.47–5.29). Participants suffering depressive symptoms were less likely to feel sufficiently prepared for SARS-CoV-2 (OR 0,51; 95%IC 0.35–0.75) and less likely to feel protected by individual protective equipment (OR 0.52; 95%CI 0.35–0.77). Several SARS-CoV-2-related stressors increased the likelihood of suffering depressive symptoms: shortfall of colleagues (OR 1.78; 95%CI 1.21–2.64), not being able to let patients down (OR 1.55; 95%CI 1.05–2.28), uncertainty about how to act correctly (OR 2.43; 95%CI 1.57–3.75), uncertainty about contact persons (OR 2.28; 95%CI 1.54–3.36), uncertainty about financial situation (OR 1.58; 95%CI 1.02–2.45), and uncertainty about temporal scope (OR 2.09; 95%CI 1.10–4.01).
George et al. [53] 2020	Objective: To describe the initial dilemmas, mental stress, and coping strategies used by HPs during the 40 first days of the COVID-19 pandemic as well as the ways in which this situation can be collectively confronted. Design: Qualitative ethnographic with an interpretative focus. Participants: A healthcare team of physicians, nurses, and paramedical and support staff, responsible for health care in a Bangalore slum. Setting: Bangalore (India). Procedure—Data collection: Semi-structured in-depth interviews, followed by focal discussion groups, carried out by 2 interviewers with previous experience in qualitative studies. Intentional and convenience sampling method was used.	There were 10 in-depth interviews (4 females, 4 males; 4 physicians, 2 nurses, 2 nurses aids, 1 lab technician, 1 driver, 1 field counselor) and 4 focal discussion groups (Group 1: 6 females, 4 males; 10 physicians; Group 2: 6 females, 3 males; 5 nurses, 4 allied health professionals; Group 3: 11 males; 9 community health workers, 2 social workers; Group 4: 2 females, 6 males; 6 drivers, 2 housekeeping staff). Stress related to the COVID-19 pandemic, the fear of death, the feeling of guilt because of infecting your loved ones, anxiety over likely violence, the stigma in marginal neighborhoods, and the exhaustion of professionals emerged as the main stress-provoking topics. The HPs used multiple adaptative interventions and coping strategies to reduce the level of anxiety and to promote the perception of self-efficacy: some centered on the emotions to favor cognitive re-evaluation and ‘positive reframing’; others focused on the problem of reducing the risk of infection; and others centered on meaning so that the stressful experience could help to maintain a sense of well-being in difficult times.
Ilczak et al. [54] 2021	Objective: To examine the professional and sociodemographic factors related to the levels of stress among Polish emergency medical personnel during the COVID-19 pandemic. Design: Descriptive cross-sectional. Participants: Physicians, nurses, and paramedics working in ambulance services and hospital emergency departments. Setting: Poland. Data collection: Online survey distributed through social networks and websites of the institutions involved in the study between 27 March and 20 April 2020. Main outcomes—Instruments: Acute stress—ad-hoc questionnaire on stressful factors	*n* = 995 HPs, of whom 565 worked on the ambulance team. No statistically significant differences were observed in the perception of occupational stress, depending on age and work experience. Women working in the EMS defined their levels of stress as significantly higher than men in the same profession (*p* < 0.001). Nurses experienced a statistically significant higher level of stress at work than paramedics during the COVID-19 pandemic (*p* = 0.009). However, no statistically significant differences were observed between paramedics and physicians or between nurses and physicians. The fear of contracting COVID-19 (β = 0.474; *p* < 0.001), a decrease in the level of safety and security while conducting emergency medical procedures (β = −0.149; *p* < 0.001), and the marginalization of patients not suffering from COVID-19 (β = −0.067; *p* = 0.014) were the main predictors of occupational stress.
Karasu el al. [55] 2021	Objective: To describe anxiety levels among Turkish HPs due to the COVID-19 pandemic. Design: Descriptive cross-sectional. Participants: HPs working at any medical institution. Setting: Turkey. Data collection: Online survey distributed through social networks between 18–25 April 2020. Main outcomes—Instruments: Anxiety—STAI.	*n* = 710 HPs, of whom 31 were first-aid and emergency-aid technicians. No statistically significant differences were observed on the subscales state and trait of anxiety among the participants in accordance with their professional category and field of work.
Maiorano et al. [56] 2020	Objective: To identify the mediating effect of the coping and stress-resistance strategies that the HPs used during the COVID-19 pandemic, which can reduce the development of symptoms of secondary trauma. Design: Descriptive cross-sectional. Participants: Workers delivering first-aid and emergency aid in different sectors during the COVID-19 pandemic. Setting: Italy Data collection: Online survey distributed through social networks, email, and online discussion groups during the first phase of forced home confinement. Main outcomes—Instruments: Acute stress—ESQ, ad-hoc questionnaire on stressful factors; Post-traumatic stress—STSS-I; Self-efficacy—CSES-EF.	*n* = 240 participants, of whom 140 were active in the Healthcare and Medical Staff group (95 females, 45 males; aged 42.03 ± 11.43 years; 74 physicians, 66 nurses; 66% treated COVID-19 patients directly), and 100 were in the Emergency group (46 females, 54 males; aged 44.80 ± 10.35 years; emergency workers, firefighters, Civil Protection staff; 54% treated COVID-19 patients directly). The Emergency group workers recorded lower levels of total stress than the professionals forming part of the Medical Staff group (69.58 ± 13.78 versus 88.62 ± 15.61, *p* < 0.05, η^2^ = 0.062), reducing the risk of developing symptoms of secondary trauma. The Emergency group workers who had been in direct contact with COVID-19 infected patients had slightly higher scores for total stress (*p* < 0.05, η^2^ = 0.024). The type of working group showed an effect between the respondents at total stress levels (*p* < 0.01, η^2^ = 0.063) and in the “stop unpleasant emotions and thoughts” strategy (*p* < 0.01, η^2^ = 0.051). Belonging to the Emergency group played a protective role against total stress (β = 2.407, *p* < 0.01).
Martínez-Caballero et al. [57] 2021	Objective: To determine the impact of the early stages of the COVID-19 pandemic on the mental health of two Spanish EMS. Design: Descriptive cross-sectional. Participants: EMS workers (physicians, nurses, and emergency medical technicians) over 18 years old, working during the COVID-19 pandemic and capable of speaking Spanish. Setting: Spain. Data collection: Online survey distributed through corporate email from 20 May to 26 July 2020. Main outcomes—Instruments: Post-traumatic stress—DTS-8; Psychological distress—GHQ-12.	*n* = 317 HPs (147 females, 167 males, 3 others; age: 136 were between 40 and 49 years old; 61 physicians, 78 nurses, 178 emergency medical technicians; 160 worked in advanced life support; 223 changed their care functions during the pandemic). Anxiety-related symptoms were experienced by 65.5% of HPs during the pandemic. Regarding psychological distress, 37.5% showed no pathology, 26.5% showed possible psychological pathology, and 36.0% showed signs of psychological pathology. In relation to post-traumatic stress, 30.9% were suspected of this disorder. Psychological health and risk of post-traumatic stress disorder were statistically related to gender (*p* < 0.001; *p* < 0.001), changes in job functions (*p* = 0.015; *p* = 0.012), having had prior theorical (*p* = 0.016; *p* = 0.008) and practical (*p* = 0.007; *p* = 0.006) training of the use of personal protective equipment, the type of SARS-CoV-2 test (*p* = 0.011; *p* = 0.001), having had adequately protective personal equipment (*p* < 0.001; *p* < 0.001), having been worried about contracting the disease (*p* < 0.001; *p* < 0.001), anxiety symptoms prior to (*p* = 0.002; *p* = 0.019) and during (*p* < 0.001; *p* < 0.001) the pandemic, use of anxiolytics during the pandemic (*p* < 0.001; *p* < 0.001), requiring psychological support prior (*p* = 0.029; *p* = 0.001) and during (*p* < 0.001; *p* < 0.001 ) the pandemic, and dealing with mental health issues normally in the work unit (*p* = 0.017; *p* = 0.012). Indeed, greater work experience (*p* = 0.05), having required isolation (*p* = 0.016), having experienced symptoms of the disease (*p* = 0.017), having had personal protective equipment removed from the service because it was not adequate (*p* = 0.026), having been worried about transmitting the disease to family (*p* < 0.001) and use of anxiolytics prior to the pandemic (*p* = 0.009) were the factors related specifically to psychological health.
Munawar et al. [58] 2021	Objective: To understand how HPs have responded to the COVID-19 pandemic, identifying possible protection factors and coping strategies used. Design: Qualitative with thematic analysis. Participants: First-line emergency HPs who had treated cases of COVID-19 in person. Setting: Pakistan. Procedure—Data collection: Semi-structured in-depth face-to-face interviews, carried out by 1 interviewer, a doctor in psychology, with previous experience in qualitative studies, between 2–25 April 2020. Convenience sampling was used to recruit participants through various social networks.	*n* = 15 participants (15 males; aged 31.87 ± 2.82 years; work experience 6.53 ± 2.54 years; 12 emergency ambulance technicians, 3 emergency ambulance drivers). The main topics and subtopics that arose from the data analysis were: 1—Stress coping mechanisms (limiting media exposure, limited sharing of COVID-19 duty details, religious coping, it is just another emergency/line of duty, altruism/empathy); 2—Challenges/Issues (psychological response, non-compliance of public/denial by religious scholars). The HPs acknowledged feelings of fear, stress, and/or anxiety as a consequence of the COVID-19 pandemic. To counter them, they practiced different coping strategies, religion and their passion for serving their community and country being the most frequently used strategies. The majority of them concluded that mass media were a source of stress and anxiety, increasing their uncertainty over the pandemic.
Skoda et al. [59] 2020	Objective: To describe the psychological burden of HPs after the COVID-19 pandemic. Setting: Descriptive cross-sectional. Participants: HPs (physicians, nurses, paramedics) and non-HPs. Setting: Germany. Data collection: Online survey distributed through the main official channels between 10 and 31 March 2020. Main outcomes—Instruments: Anxiety—GAD-7; Depression—PHQ-2.	*n* = 2224 HPs, of whom 221 were paramedics (55 females, 164 males, 2 diverse individuals). Among these paramedics, 14 suffered a mental illness; 50 had risk factors consistent with a severe case of COVID-19). The lowest levels of generalized anxiety were observed among paramedical staff; 4.55% of them presented moderate-to-high levels (GAD-7 ≥ 10). Believing to be sufficiently well informed about COVID-19 reduced the levels of generalized anxiety (PR 0.833, 95%IC 0.24–2.80) Paramedics presented lower levels of depression than the non-HPs (*d* = 0.285, 95%CI 0.152–0.418), with neither physicians nor nurses showing any observable differences.
Sorokin et al. [60] 2020	Objective: To evaluate the structure and the seriousness of stress and stigmatization among different HPs during the COVID-19 pandemic. Setting: Descriptive cross-sectional. Participants: HPs over 18 years old, capable of reading and understanding texts written in Russian. Setting: Russia. Data collection: Online survey distributed through social networks and topical websites from 30 March to 5 April and 4–10 May 2020. Main outcomes—Instruments: Acute stress—PSM-25.	*n* = 1800 HPs (1459 females, 341 males; aged 42 ± 12 years; work experience 17 ± 12 years), of whom 63 were paramedic staff. Physicians suffered higher stress levels than nurses (*d* = −0.34) and paramedical staff (*d* = −0.64).
Torrente et al. [61] 2021	Objective: To evaluate the prevalence of burnout syndrome among HPs during the COVID-19 pandemic, identifying differences between those who worked on the front line and those who did so in their normal jobs (before the pandemic). Design: Descriptive cross-sectional. Participants: Physicians, nurses, nursing assistants, and emergency medical technicians working during the COVID-19 pandemic. Setting: Spain. Data collection: Online survey distributed by email and social media between 21 April and 3 May 2020. Main outcomes—Instruments: Burnout—MBI.	*n* = 643 HPs, of whom 128 had out-of-hospital EMS as an area of work (101 in the front line and 27 in other posts). No statistically significant differences were observed in the levels of burnout nor on the psychological impact of COVID-19 on HPs as a function of professional category and area of work.
Usul et al. [62] 2020	Objective: To identify the factors related with the level of anxiety among EMS during the COVID-19 pandemic. Design: Descriptive cross-sectional. Participants: EMS professionals. Setting: Ankara (Turkey). Data collection: Personal interview with each participant. Main outcomes—Instruments: Anxiety—STAI Subscale State.	*n* = 402 EMS professionals (190 females, 212 males; aged 33.1 ± 6.9 years; work experience 8.9 ± 5.4 years; 110 paramedics, 208 emergency medical technicians, 14 nurses, 20 physicians, 50 drivers; 10 had a previous or an existing mental disorder; 83.8% treated COVID-19 patients directly). The mean anxiety score was 50.7 ± 11.6, being higher among women (53.9 ± 10.5 vs. 47.8 ± 11.9, *p* <0.001). A reduction in anxiety levels was observed as the age of the participants increased (*p* < 0.05). Nurses had a significantly lower score (41.2 ± 13.8) than paramedics (52.0 ± 11.6), emergency medical technicians (51.7 ± 11.1), and physicians (43.9 ± 12.7) (all *p* < 0.001). Being concerned over infecting family members, thinking that appropriate protective equipment is not available, not feeling sure or feeling more nervous when treating possible or confirmed cases of COVID-19, or feeling more nervous in general were related to significantly higher levels of anxiety (all *p* < 0.01).
Vagni et al. [63] 2020	Objective: To identify the coping strategies used among HPs to confront the stress factors arising from the COVID-19 pandemic that contributed to reducing the inductive factors of secondary trauma symptoms. Design: Descriptive cross-sectional Participants: HPs and emergency workers active in different sectors during the COVID-19 pandemic. Setting: Italy. Data collection: Online survey distributed through social networks, email, and on-line discussion groups during forced home confinement. Main outcomes—Instruments: Acute stress—ESQ, ad hoc questionnaire on stressful factors; Post-traumatic stress—STSS-I, Self-efficacy—CSES-SF.	*n* = 210 participants, of whom 121 were in the Health group (80 female, 41 male; aged 42.13 ± 11.35 years; work experience 14.60 ± 11.56 years; 58 physicians, 47 nurses, 9 psychologists, 7 healthcare assistants; 73% treated COVID-19 patients directly) and 89 in the Emergency group (40 females, 49 males; aged 45.43 ± 10.19 years; work experience 14.41 ± 11.89 years; 48 emergency workers, 21 firefighters, 20 Civil Protection staff; 33% treated COVID-19 patients directly). No statistically significant differences were observed in the stress levels, symptoms of secondary trauma, and coping strategies among workers from the Emergency group. In comparison with the Health group, the professionals included in the Emergency group experienced lower degrees of physiological and psychological activation (26.33 ± 4.97 vs. 23.30 ± 5..51, *d* = 0.58, *p* < 0.001), organizational–relational stress (22.69 ± 4.43 vs. 19.43 ± 3.62, *d* = 0.81, *p* < 0.001), physical stress (10.29 ± 3.13 vs. 8.09 ± 4.60, *d* = 0.45, *p* < 0.01), inefficacy decision stress (14.45 ± 3.13 vs. 12.79 ± 3.05, *d* = 0.54, *p* < 0.001), emotional stress (14.17 ± 3.48 vs. 10.45 ± 3.16, *d* = 1.12, *p* < 0.001), cognitive stress (8.88 ± 2.89 vs. 6.08 ± 2.53, *d* = 1.03, *p* < 0.001), and COVID-19 stress (15.54 ± 3.67 vs. 12.74 ± 4.17, *d* = 0.71, *p* < 0.001). Stopping unpleasant emotions and thoughts was the coping strategy most frequently used by the workers from the Emergency group (32.50 ± 10.79 vs. 36.40 ± 9.00, *d* = 0.40, *p* < 0.01). Belonging to the Emergency group was a protective factor against organizational relational stress (β = −0.197, *p* < 0.01), inefficacy–decisional stress (β = −0.175, *p* < 0.05), emotional stress (β = −0.351, *p* < 0.001), cognitive stress (β = −0.316, *p* < 0.001), COVID-19 stress (β = −0.283, *p* < 0.001), and arousal as well as the degree of physiological and psychological activation as a symptom of secondary stress (β = −0.264, *p* < 0.001).
Vagni et al. [64] 2020	Objective: To identify the resistance skills of HPs in confronting the stress caused by the COVID-19 pandemic, associated with the risk of developing symptoms of secondary trauma. Design: Descriptive cross-sectional. Participants: Red Cross volunteers. Setting: Véneto (Italy). Data collection: Online survey distributed through social media, email, and online discussion groups during the second phase of the pandemic. Main outcomes—Instruments: Acute stress—ESQ, ad hoc questionnaire on stressful factors; Burnout—MBI.	*n* = 494 volunteers (280 females, aged 44.40 ± 12.92 years; 214 males, aged 47.52 ± 13.52 years; weekly work 13.05 ± 12.26 hours), of whom 186 were included in the Health group (emergency room interventions and transport of the sick), 151 in the Social group (social support and inclusion actions), and 157 in the Emergency group (management of emergency and COVID-19 units). All the subscales of stress showed a positive correlation with emotional exhaustion and depersonalization and negative correlation with personal accomplishment. In the Emergency group, the older HPs showed higher levels of organizational–relational stress (*p* < 0.05, η^2^ = 0.011), physical stress (*p* < 0.01, η^2^ = 0.017), and emotional stress (*p* < 0.01, η^2^ = 0.019) as well as lower personal accomplishment (*p* < 0.01, η^2^ = 0.016). Among them, those who directly treated patients with COVID-19 reported greater inefficacy–decisional stress (*p* < 0.001, η^2^ = 0.038) and personal accomplishment (*p* < 0.001, η^2^ = 0.028). The type of working group showed an effect between the individuals at the level of COVID-19 stress *(p* < 0.05, η^2^ = 0.018) and personal accomplishment (*p* < 0.05, η^2^ = 0.015).
Vagni et al. [65] 2020	Objective: To identify the capabilities of HPs to resist COVID-19 pandemic-related stress, associated with the risk of developing symptoms of secondary trauma. Design: Descriptive cross-sectional. Participants: HPs and emergency workers. Setting: Italy. Data collection: Online survey distributed during the pandemic. Main outcomes—Instruments: Acute stress—ESQ; Post-traumatic stress—STSS-I.	*n* = 236 participants (139 females, 97 males; aged 43.24 ± 11.06; 56.8% treated COVID-19 patients directly), of whom 140 were included in the Health group (44 females, 95 males; 64 physicians, 55 nurses, 11 operators, 10 psychologists; 72.9% treated COVID-19 patients directly) and 96 in the Emergency group (52 females, 45 males; 51 ambulance workers, 45 other operators such as firefighters, police or Civil Protection staff; 33.3% treated COVID-19 patients directly). No statistically significant differences were observed at the levels of stress or symptoms of secondary trauma in the workers of the Emergency group. In comparison with the HPs from the Healthcare group, those included in the Emergency Group presented lower levels of total stress (84.34 ± 15.01 vs. 69.69 ± 19.02, *d* = 1.09, *p* < 0.01), organizational–relational stress (22.04 ± 4.69 vs. 19.32 ± 3.61, *d* = 0.65, *p* < 0.01), physical stress (10.09 ± 5.24 vs. 8.07 ± 4.48, *d* = 0.41, *p* < 0.05), inefficacy–decisional stress (14.72 ± 2.47 vs. 12.77 ± 2.47, *d* = 0.79, *p* < 0.01), emotional stress (13.90 ± 3.58 vs. 10.58 ± 3.49, *d* = 0.90, *p* < 0.01), cognitive stress (8.65 ± 2.89 vs. 6.10 ± 2.34, *d* = 0.94, *p* < 0.01), COVID-19 stress (15.18 ± 3.49 vs. 12.85 ± 4.09, *d* = 0.61, *p* < 0.01), as well as the degree of physiological and psychological activation (26.48 ± 4.04 vs. 23.69 ± 4.27, *d* = 0.67, *p* < 0.01). The women from the Emergency group reported major physical stress (9.41 ± 4.70 vs. 6.94 ± 3.99, *p* < 0.01) and emotional stress (11.52 ± 3.94 vs. 9.79 ± 2.86, *p* < 0.05); whereas the men obtained greater inefficacy–decisional stress (13.37 ± 2.34 vs. 12.07 ± 2.46, *p* < 0.05). No significant differences were observed for stress levels among HPs as a function of direct assistance to patients with COVID-19 or otherwise. Not belonging to the Emergency group was a risk factor of an increased degree of physiological and psychological activation as well as a symptom of secondary trauma (β = −0.338, *p* < 0.001).
Vagni et al. [66] 2020	Objective: To identify the mediating effect of resistance and the coping strategies activated by emergency services workers to withstand the stress and symptoms of secondary trauma caused by the COVID-19 pandemic. Design: Descriptive cross-sectional. Participants: Red Cross Emergency volunteers. Setting: Venice (Italy). Data collection—Instrument: Online survey distributed during forced home confinement. Main outcomes—Instruments: Acute stress—ESQ; Post-traumatic stress—STSS-I; Self-efficacy—CSES-SF.	*n* = 513 volunteers (286 females, aged 44.49 ± 12.99 years; 227 males, aged 47.10 ± 13.51 years; weekly work 13.49 ± 11.62 h). All subscales of stress showed a positive correlation with secondary trauma and a negative one with coping strategies. The women presented significantly higher scores for physical stress (6.88 ± 4.83 vs. 4.86 ± 4.53, *p* < 0.001), emotional stress (8.33 ± 4.18 vs. 7.41 ± 4.69, *p* < 0.05), degree of physiological and psychological activation (11.86 ± 3.99 vs. 9.84 ± 4.17, *p* < 0.001), avoidance behavior (12.70 ± 3.56 vs. 11.47 ± 3.89, *p* < 0.001) and obsessive thoughts (9.69 ± 3.14 vs. 8.86 ± 3.28, *p* < 0.01), whereas the men scored significantly higher in problem-focused coping strategies (39.00 ± 6.42 vs. 37.82 ± 6.49, *p* < 0.05). Weekly hours of work had no effect on the stress factors, the symptoms of secondary trauma, and the coping strategies. Being a woman was a predictive factor of physical stress (β = 0.383, *p* < 0.001), degree of physiological and psychological activation (β = 0.890, *p* < 0.001), avoidance behavior (β = 0.508, *p* < 0.05), and obsessive thoughts (β = 0.464, *p* < 0.05). Age was a predictive factor of physical stress (β = −0.117, *p* < 0.01), organizational–relational stress (β = −0.105, *p* < 0.05), emotional stress (β = −0.102, *p* < 0.05), cognitive stress (β = −0.091, *p* < 0.05), avoidance behavior (β = 0.032, *p* < 0.01), and obsessive thoughts (β = 0.049, *p* < 0.001). Having no personal protection equipment was a predictive factor of organizational–relational stress (β = 0.304, *p* < 0.001), physical stress (β = 0.116, *p* < 0.01), emotional stress (β = 0.129, *p* < 0.01), cognitive stress (β = 0.233, *p* < 0.001), and avoidance behavior (β = 0.081, *p* < 0.05). Stopping unpleasant emotions and thoughts coping strategy was a predictive factor of organizational–relational stress (β = −0.156, *p* < 0.01), physical stress (β = −0.332, *p* < 0.001), emotional stress (β = −0.353, *p* < 0.001), cognitive stress (β = −0.244, *p* < 0.001), COVID-19 stress (β = −0.252, *p* < 0.001), degree of physiological and psychological activation (β = 0.039, *p* < 0.05), and obsessive thoughts (β = 0.032, *p* < 0.05). Supportting a coping strategy was a predictive factor of avoidance behavior (β = 0.057, *p* < 0.05). Organizational–relational stress was a predictive factor of obsessive thoughts (β = −0.079, *p* < 0.05), physical stress, degree of physiological and psychological activation (β = 0.334, *p* < 0.001); avoidance behavior (β = 0.147, *p* < 0.001) and obsessive thoughts (β = 0.122, *p* < 0.001); inefficacy–decisional stress, of obsessive thoughts (β = 0.064, *p* < 0.05); emotional stress, of the degree of physiological and psychological activation (β = 0.270, *p* < 0.001), avoidance behavior (β = 0.222, *p* < 0.001), and obsessive thoughts (β = 0.189, *p* < 0.001); cognitive stress, of avoidance behavior (β = 0.237, *p* < 0.001) and obsessive throughts (β = 0.129, *p* < 0.05); and COVID-19 stress, degree of physiological and psychological activation (β = 0.127, *p* < 0.001), avoidance behavior (β = 0.078, *p* < 0.05), and obsessive thoughts (β = 0.254, *p* < 0.001). Total stress had predictive effects on the degree of physiological and psychological activation (β = 0.698, *p* < 0.001) and avoidance behavior (β = −0.391, *p* < 0.001); in both cases, problem-focused and “stop unpleasant emotional thoughts” coping strategies acted as mediating factors.
Vagni et al. [67] 2021	Objective: To examine the relationships between emergency stress and coping strategies in HPs and compare the results of the first and second waves. Design: Descriptive cross-sectional. Participants: HPs and emergency workers active in different sectors during the COVID-19 pandemic. Setting: Italy. Data collection—Instrument: Online survey distributed during the forced home confinement on April 2020 and during the second pandemic wave between November and December 2020. Main outcomes—Instruments: Acute stress—ESQ, PSS; Post-traumatic stress—STSS-I; Burnout—MBI; Self-efficacy—CSES-SF.	*n* = 500 participants. In the first wave, 140 were included in the Health group (74 physicians, 66 nurses) and 100 in the Emergency group (100 emergency workers); in the second wave, 179 HPs were included in the Health group (50 physicians, 129 nurses) and 81 in the Emergency group (81 emergency workers); 63.7% and 72.3% worked with COVID-19 patients on the first and second waves, respectively. The total stress levels of the Emergency group did not differ between the two waves of the pandemic; physical stress was greater in the second wave. No differences were observed in the coping strategies used by the participants. An analysis of burnout levels in the second wave found that total stress levels showed a high predictive power in the emotional exhaustion (β = 0.62; *p* < 0.001) and depersonalization (β = 0.20; *p* < 0.001) subscales.
Vanhaecht et al. [68] 2021	Objective: To determine the effect of COVID-19 on the positive or negative symptoms of mental health of HPs, identifying sources of support from the workplace authorities. Design: Descriptive cross-sectional. Participants: HPs working during the week before the completion of the questionnaire. Setting: Flanders (Belgium). Data collection: Online survey distributed through social networks between 2 April and 4 May 2020. Main outcomes—Instruments: Mental health symptoms—frequency of 19 negative and positive signs and symptoms of mental health (ad hoc).	*n* = 4503 HPs, of whom 1831 were paramedics. A greater occurrence of negative symptoms was observed among the paramedics during the COVID-19 pandemic in relation to the previous situation: stress (OR 5.42, 95%CI 4.79–6.13), hypervigilance (OR 10.95, 95%CI 9.59–12.51), fatigue (OR 5.15, 95%CI 4.56–5.83), difficulty sleeping (OR 6.04, 95%CI 5.33–6.84), unable to relax (OR 4.24, 95%CI 3.75–4.80), fear (OR 9.88, 95%CI 8.66–11.27), irregular lifestyle (OR 4.06, 95%CI 3.56–4.05), difficulty concentrating (OR 4.33, 95%CI 3.81–4.92), unhappy and dejected (OR 4.96, 95%CI 4.35–5.65), failure to recognize own emotional response (OR 3.88, 95%CI 3.40–4.44), doubting knowledge and skills (OR 2.90, 95%CI 2.57–3.28), feeling on their own (OR 3.16, 95%CI 2.78–3.58), avoiding risks (OR 4.31, 95%CI 3.77–4.91), leaving the profession (OR 2.47, 95%CI 2.15–2.85), and uncomfortable within a team (OR 2.59, 95%CI 2.28–2.95). Positive symptoms such as forming part of a team (OR 0.69, 95%CI 0.61–0.78), making a difference (OR 0.80, 95%CI 0.71–0.91), and sufficient support guidance (OR 0.74, 95%CI 0.66–0.84) were less likely to be present within this group during the COVID-19 pandemic in relation to the previous situation. Likewise, a significant modification of the effect was observed according to professional category since the ORs for all symptoms were lower in paramedics relative to nurses.
Vujanovic et al. [69] 2021	Objective: To evaluate the impact of exposure to COVID-19 on the mental health of first-line healthcare workers. Design: Descriptive cross-sectional. Participants: First-line healthcare workers older than 18 years in age. Setting: United States. Data collection: Online survey distributed by email between June and August 2020. Main outcomes—Instruments: Anxiety—OASIS; Depression—ODSIS; Post-traumatic stress—PCL-5 adapted to COVID-19; Mental health—MHCQ.	*n* = 189 first aid responders (40 females, 149 males; aged 47.6 ± 10.9 years; 60 firefighter, 35 EMS, 91 firefighters—EMS, 3 firefighters—EMS law enforcement). In all, 6.8% fitted the clinical criteria for post-traumatic stress, 18.4% for anxiety, and 16.8% for depression. Among the first-aid responders, those who reported exposure to COVID-19 (*n* = 122) had a high consumption of alcohol (*p* = 0.016), worked in an EMS (*p* = 0.03), or had obtained a recognized qualification (*p* = 0.04). Their mean level of anxiety was 4.57 ± 0.36 and their mean level of depression was 3.83 ± 0.38, observing no statistically significant differences in both variables as a function of exposure to COVID-19 or otherwise (*p* > 0.05). A greater concern for COVID-19 was related with higher levels of anxiety (β = 0.52, *p* < 0.001), depression (β = 0.34; *p* < 0.001), or presence of post-traumatic stress symptoms (β = 0.71, *p* < 0.001). A greater medical vulnerability was related to higher levels of anxiety (β = 0.16, *p* < 0.012) or depression (β = 0.25, *p* < 0.012). A shorter working life was related to higher levels of depression (β = −0.18, *p* < 0.013).
Zolnikov et al. [70] 2020	Objective: To understand the stigma and consequences for the mental health of first-aid responders during the COVID-19 pandemic. Design: Qualitative with a phenomenological focus. Participants: HPs and first-aid responders, older than 18 years, who had worked during the COVID-19 pandemic. Setting: Canada, United States, Ireland, Kenya. Procedure—Data collection: Semi-structured interviews conducted over the telephone. Convenience sampling was used to select participants through different social networks.	*n* = 31 participants (18 females, 13 males; aged 36.13 years; 3 physicians, 14 nurses, 1 nurse tech, 1 behavioral therapist, 1 orthodontist, 1 dialysis technician, 1 technician in medical surgery, 1 data specialist, 1 paramedic, 3 firefighters and paramedics, 1 firefighter and medical emergency technician, 3 police officers). The first-aid responders expressed feelings of isolation and the lack of support and understanding among family and friends, reduction or forced removal in immediate social interactions, feelings of being infected or dirty, increased feelings of sadness and anxiety, and refusal to seek help or to receive treatment.

HP: healthcare professional; COVID-19: coronavirus disease; EMS: Emergency Medical Services; GAD-2: Generalized Anxiety Disorder 2-items; PHQ-2: Patient Health Questionnaire 2; OR: odds ratio; STAI: State-Trait Anxiety Inventory; ESQ: Emergency Stress Questionnaire; STSS-I: Secondary Traumatic Stress Scale—Italian version; CSES-EF: Coping Self-Efficacy Scale—Short Form; DTS-8: Davidson Trauma Scale; GHQ-12: General Health Questionnaire; GAD-7: Generalized Anxiety Disorder 7; PR: prevalence ratio; CI: confidence interval; PSM-25: Psychological Stress Measure; MBI: Maslach Burnout Inventory; PSS: Perceived Stress Scale; OASIS: Overall Anxiety Severity and Impairment Scale; ODSIS: Overall Depression Severity and Impairment Scale: PCI-5: Post-traumatic Stress Disorder Checklist for DSM-5, adapted to COVID-19; MHCQ: Mental Health Correlates Questionnaire.

**Table 3:** Critical appraisal of cross-sectional studies.

Citation	Q1	Q2	Q3	Q4	Q5	Q6	Q7	Q8
Dreher et al. [52]	Y	Y	Y	N	Y	Y	N	Y
Ilczak et al. [54]	Y	Y	Y	N	Y	Y	Y	Y
Karasu et al. [55]	Y	Y	Y	Y	N	N	Y	Y
Maiorano et al. [56]	Y	Y	Y	N	Y	Y	N	Y
Martinez-Caballero et al. [57]	Y	Y	Y	Y	N	N	Y	Y
Skoda et al. [59]	Y	Y	Y	Y	N	N	Y	Y
Sorokin et al. [60]	Y	Y	Y	Y	N	N	Y	Y
Torrente et al. [61]	Y	Y	N	N	Y	Y	Y	Y
Usul et al. [62]	Y	Y	Y	Y	N	N	Y	Y
Vagni et al. [63]	Y	Y	Y	N	Y	Y	Y	Y
Vagni et al. [64]	Y	Y	Y	N	Y	Y	Y	Y
Vagni et al. [65]	Y	Y	Y	N	Y	Y	Y	U
Vagni et al. [66]	Y	Y	N	N	Y	Y	Y	Y
Vagni et al. [67]	Y	Y	Y	N	Y	Y	Y	Y
Vanhaedad et al. [68]	Y	Y	N	N	Y	Y	Y	Y
Vujanovic et al. [69]	Y	Y	N	N	Y	Y	Y	Y
% Y	100.00	100.00	75.00	45.00	75.00	75.00	90.00	90.00

Y: yes; N: no; U: unclear. Q1: Were the criteria for inclusion in the sample clearly defined? Q2: Were the study subjects and the setting described in detail? Q3: Was the exposure measured in a valid and reliable way? Q4: Were objective, standard criteria used for measurement of the condition? Q5: Were confounding factors identified? Q6: Were strategies to deal with confounding factors stated? Q7: Were the outcomes measured in a valid and reliable way? Q8: Was appropriate statistical analysis used?

**Table 4:** Critical appraisal of qualitative studies.

Citation	Q1	Q2	Q3	Q4	Q5	Q6	Q7	Q8	Q9	Q10
Arbedilli et al. [51]	Y	Y	Y	Y	Y	Y	N	N	Y	Y
George et al. [53]	U	Y	Y	Y	Y	Y	Y	Y	U	Y
Munawar et al. [58]	Y	Y	Y	Y	Y	Y	U	Y	U	Y
Zolkinov et al. [70]	Y	Y	Y	Y	Y	Y	Y	Y	Y	Y
% Y	75.00	100.00	100.00	100.00	100.00	100.00	50.00	75.00	50.00	75.00

Y: yes; N: no; U: unclear. Q1: Is there congruity between the stated philosophical perspective and the research methodology? Q2: Is there congruity between the research methodology and the research question or objectives? Q3: Is there congruity between the research methodology and the methods used to collect data? Q4: Is there congruity between the research methodology and the representation and analysis of data? Q5: Is there congruity between the research methodology and the interpretation of results? Q6: Is there a statement locating the researcher culturally or theoretically? Q7: Is the influence of the researcher on the research, and vice-versa, addressed? Q8: Are participants and their voices adequately represented? Q9: Is the research ethical according to current criteria or for recent studies, and is there evidence of ethical approval by an appropriate body? Q10: Do the conclusions drawn in the research report flow from the analysis or interpretation of the data?

## Data Availability

Data for this study are available by contacting the corresponding author.
